# A systematic review of causes of sudden and severe headache (Thunderclap Headache): should lists be evidence based?

**DOI:** 10.1186/1129-2377-15-49

**Published:** 2014-08-14

**Authors:** Emma Devenney, Hazel Neale, Raeburn B Forbes

**Affiliations:** 1 Department of Neurology and Medical Library, Craigavon Area Hospital, Southern HSC Trust, County Armagh, Northern Ireland BT63 5QQ, UK

**Keywords:** Thunderclap headache, Sudden headache, Acute headache, Systematic review, Publication bias

## Abstract

**Background:**

There are many potential causes of sudden and severe headache (thunderclap headache), the most important of which is aneurysmal subarachnoid haemorrhage. Published academic reviews report a wide range of causes. We sought to create a definitive list of causes, other than aneurysmal subarachnoid haemorrhage, using a systematic review.

**Methods:**

Systematic Review of EMBASE and MEDLINE databases using pre-defined search criteria up to September 2009. We extracted data from any original research paper or case report describing a case of someone presenting with a sudden and severe headache, and summarized the published causes.

**Results:**

Our search identified over 21,000 titles, of which 1224 articles were scrutinized in full. 213 articles described 2345 people with sudden and severe headache, and we identified 6 English language academic review articles. A total of 119 causes were identified, of which 46 (38%) were not mentioned in published academic review articles. Using capture-recapture analysis, we estimate that our search was 98% complete. There is only one population-based estimate of the incidence of sudden and severe headache at 43 cases per 100,000. In cohort studies, the most common causes identified were primary headaches or headaches of uncertain cause. Vasoconstriction syndromes are commonly mentioned in case reports or case series. The most common cause not mentioned in academic reviews was pneumocephalus. 70 non-English language articles were identified but these did not contain additional causes.

**Conclusions:**

There are over 100 different published causes of sudden and severe headache, other than aneurysmal subarachnoid haemorrhage. We have now made a definitive list of causes for future reference which we intend to maintain. There is a need for an up to date population based description of cause of sudden and severe headache as the modern epidemiology of thunderclap headache may require updating in the light of research on cerebral vasoconstriction syndromes.

## Background

Sudden and Severe Headache is a common reason for seeking urgent medical advice [1], primarily to exclude aneurysmal subarachnoid haemorrhage [2].

In the process of developing a guideline for managing acute headache we observed variation in the range of diagnoses published [3-9], so we conducted a systematic review of literature to compile a comprehensive list of potential causes other than aneurysmal subarachnoid haemorrhage.

We estimate completeness of ascertainment of causes using capture-recapture methods, and discuss the implications of using systematic review methods for lists of causation, paying attention to the issue of publication bias.

## Methods

We searched MEDLINE and EMBASE databases in September 2009 using pre-specified search criteria (Table 1), designed and implemented by a professional librarian (HN), as the Medical Subject Heading (MeSH term) for Thunderclap Headache excludes articles indexed prior to November 1999 [10]. We screened titles of publications for potential cases, case series, cohort studies or academic reviews of people with a sudden AND severe headache. Cases were included if the presenting headache was both sudden in onset AND severe in intensity. Cases were excluded if a headache onset was not sudden or if onset was not reported (even the headache was severe or a significant aetiology was subsequently found). Similarly, if a sudden headache was described but there was no description of the severity of the pain, we excluded the case. We excluded publications concerning aneurysmal subarachnoid haemorrhage, as the major purpose of our systematic review was to identify other causes. Publications on Exploding Head Syndrome [11], Neuralgic disorders, such as Idiopathic Stabbing Headaches [12], or Trigeminal Neuralgia [13] were also excluded, as these are characteristic syndromes in their own right. However, these diagnoses could be included if they were reported as the final diagnosis in someone presenting with a sudden AND severe headache.

**Table 1:** Search criteria to identify articles on sudden and severe headache (performed September 2009)

**Ovid MEDLINE(R)**		**EMBASE**			
**Search terms**		**Search terms**	**1996-2009**	**1988-95**	**1980-87**
1. headache/di, ep, et, co, mo, cf, ra		1. exp primary headache/	165	0	0
2. headache disorders, primary/		2. exp cough headache/	15	0	0
3. vascular headaches/		3. exp exertional headache/	17	0	1
4. intracranial hypertension/		4. exp hypnic headache/	81	0	0
5. intracranial hypotension/		5. exp postural headache/	24	1	0
6. pseudotumor cerebri/		6. exp secondary headache/	43	0	0
7. intracranial vasospasm/		7. exp thunderclap headache/	84	0	9
8. (acute disease/ or catastrophic illness.mp. or critical illlness/ or emergencies/) and (headache/ or headache disorder/) [mp=title, original title, abstract, name of substance word, subject heading word]		8. exp vascular headache/	88	0	1
9. 7 and (headache/ or headache disorder/)		9. (coitus/ or orgasm/) and exp "headache and facial pain"/	118	14	7
10. thunderclap headache?.mp.		10. exp acute disease/ and exp "headache and facial pain"/	624	17	20
11. coit*.mp. and (headache/ or headache disorder/)		11. exp intracranial hypotension/	589	8	11
12. call fleming.mp.		12. exp brain pseudotumor/ and exp "headache and facial pain"/	387	60	2
13. worst headache?.mp.		13. exp brain vasospasm/ and exp "headache and facial pain"/	181	18	11
14. sentinel headache?.mp.		14. thunderclap headache?.mp.	165	11	0
15. explosive headache?.mp.		15. coit*.mp. and exp "headache and facial pain"/	61	16	1
16. reversible cerebral vaso*.mp.		16. call fleming.mp.	16	0	3
17. postpartum angio*.mp.		17. worst headache?.mp.	21	5	0
18. postpartum vasc*.mp.		18. sentinel headache?.mp.	14	3	0
19. 1 or 2 or 3 or 4 or 5 or 6 or 7 or 8 or 9 or 10 or 11 or 12 or 13 or 14 or 15 or 16 or 17 or 18		19. explosive headache?.mp.	6	1	0
		20. reversible cerebral vaso*.mp.	45	1	66
		21. postpartum angiopathy.mp.	14	0	57
Gross total references	20338	Gross total references	2758	155	57
Duplicates	1547	Duplicate references	18	14	0
Net total references from MEDLINE	18791	Net total references	2395	141	57
All references Identified					21384

We included Academic Review articles, as we wanted to compare causes published in academic reviews with those obtained from the data sourced from our systematic review. Academic Review articles had to describe thunderclap headache or the differential diagnosis of thunderclap headache, and not just headache seen in the Emergency Department. We drew up lists of causes which were (a) identified in original articles only, (b) identified in academic review articles only and (c) identified in both original articles and academic reviews.

We were unable to use the International Headache Classification [14], as the majority of authors had not used criteria, and there was usually insufficient information to allow a retrospective classification. We therefore classified cases according to causation.

### Non-English publications

We were not funded for translation services. Once we had completed the review of English publications, we used an online translation tool [15], and entered text from non-English articles to try and identify additional causes from non-English publications. As we were not able to have these papers translated professionally, we summarised these articles separately.

### Statistics and analysis

We used descriptive, summary statistics and stratified our diagnostic categories according to whether the source cases were from case reports, case series or cohort studies.

We were specifically interested in whether there was variation between different study types, as publication bias would suggest that conditions that are rare in unselected populations would be over-represented in the medical literature. We were also interested in exploring any differences between lists of causes identified by Academic Review articles and the list we ultimately generated from our systematic review.

To assess the completeness of our diagnostic list we used a capture-recapture method to estimate the number of unobserved diagnoses [16].

We were advised by our local research ethics committee that formal approval was not required as this research did not involve human subjects, and was a systematic review of published literature.

## Results

Our search strategy, executed in September 2009, identified 21,384 potential articles (Table 1). On review of titles we created a shortlist of 1224 publications. The abstracts of these 1224 full text articles were reviewed. 151 publications were duplicates (i.e. listed on both EMBASE and MEDLINE), leaving 1073 papers. Of these 1073, 70 required translation (which we did not have funding to do), and 27 were not traceable, even following inter-library loan requests.

Of the remaining 976 articles, 213 met inclusion criteria of describing a total of 2345 people with sudden and severe headache:

• 194 Case Report articles describing 321 people

• 13 Case Series articles describing 375 people

• 6 Cohort articles describing 1649 people.

Only one study had prospective, unselected population-based information on the incidence of Sudden Headache - estimating an annual incidence of 43 cases per 100,000 adults [17].

A further 6 articles were identified which were English Language Academic Review articles on Thunderclap Headache that described causes of sudden AND severe i.e. ‘Thunderclap’ headache [3,4,6,7,18,19].

This systematic review is therefore based upon a total of 219 English publications (213 original articles and 6 academic reviews). The 764 articles excluded articles were reviews related to acute headache, the investigation of headache, editorial comment or publications of new onset headache where onset AND severity were not reported.

Of the 70 non-English articles, we excluded 29 after using an online translation tool. Of the remaining 41 non-English Language articles, 22 were case reports which included 29 cases, none of which reported causes of sudden and severe headache that were unreported in our English Language papers. There were 8 case series and 4 cohort studies looking at unselected series of people with acute headache. A further 6 non-English academic review articles were identified that examined thunderclap headache [20-25].

### Completeness of ascertainment of systematic review

There were a total of 69 distinct clinical syndromes associated with Sudden AND Severe Headache that were mentioned in academic review articles AND were identified in our own literature search. We identified 46 distinct clinical syndromes associated with Sudden and Severe Headache that were unique to our systematically reviewed literature, and there were 4 diagnoses mentioned in the academic review articles as causes of Thunderclap Headache, but we did not find any citation in our own search strategy (‘Crash Migraine’, Hypnic Headache, Anterior Cerebral Artery Aneurysm (although we identified a case of middle cerebral artery aneurysm), and Cluster Headache).

If we assume that we missed these 4 causes, then our search strategy has probably identified about 98% of known causes of sudden and severe headache (Table 2).

**Table 2:** Estimated efficiency of systematic search for causes of sudden and severe headache

		**In academic review**	
		**Yes**	**No**
In systematic review	Yes	69**	46
	No	4	*Estimated 3 articles unobserved

*Where the number of unobserved cases is equal to (46 × 4) / 69 (see reference [16,26].

**If we count all vasoconstriction syndromes as one single cause, then the value of this cell in 45, but the number of unobserved cases would still be close to 3.

#### Relative frequency of causes, according to type of study

The frequency of causes depended on the type of study performed (Table 3).

**Table 3:** Sudden AND severe headache causation by group and type of publication

**Cause**	**Cohort (N)**	**%**	**Case (N)**	**%**	**Case series**	**%**	**All (N)**	**%**
Idiopathic thunderclap headache	265	16	87	27	107	29	459	20
Other, not specified	457	28	18	6	0	0	475	20
Primary headache	447	27	0	0	0	0	447	19
Cerebrovascular	281	17	153	47	198	53	632	27
Infection	119	7	17	5	24	6	160	7
Unknown	49	3	0	0	0	0	49	2
Non-neurovascular	27	2	46	14	46	12	119	5
Sudden death with headache	4	0.2	0	0	0	0	4	0
**Total**	**1,649**	**100**	**321**	**99**	**375**	**100**	**2345**	**100**

By definition, case reports and case series would not contain any people with sudden and severe headache in whom a cause was uncertain or unknown. There were no published case reports of common primary headaches, such as migraine, tension-type headache or cluster headache manifesting as sudden and severe headache (Table 3). On the other hand, Reversible Cerebral Vasoconstriction Syndromes were not explicitly mentioned in any of the published cohort studies, but were common subjects of case series and case reports.

In cohort studies (Table 3), where neurologists were responsible for assigning diagnoses, the ‘not-specified’ populations (28%) make the largest group of people. Overall, from cohort studies, 75% of cases of sudden and severe headache are assigned to either a primary headache disorder (27%), a non-specific headache (28%), Idiopathic Thunderclap Headache (17%) or an uncertain headache disorder (3%).

In cohort studies, a Cerebrovascular Disorder (17%) is most the most likely identifiable cause. Cerebral Vasoconstriction Syndromes were not explicitly reported in any of the cohort studies, but systemic or CNS infections, including sinusitis, meningitis and viral illness are reasonably frequently associated with sudden and severe headache (7%).

Cervicocephalic Arterial Dissection (0.1% of cohort cases) and Cerebral Venous Sinus Thrombosis (0.2% of cohort cases) are often cited as causes of sudden and severe headache, and featured prominently from our case series and case reports, yet occur infrequently in cohort studies. Pituitary Apoplexy was not reported in any of the cohort studies.

Other rare causes from cohort studies that were not published in case reports included Acute Cranial Herpes Zoster [27], Herpes Encephalitis [28], HaNDL Syndrome [27], Cerebral Oedema [17,29] and Alcohol-Induced Headache [27].

Case series are usually single institution experiences of diagnosis and management of a single cause of sudden and severe headache. Cerebrovascular causes predominate, such as Reversible Cerebral Vasoconstriction Syndrome [30] (Table 3). In the Case Reports, cerebrovascular causes accounted for 47% of published cases (Table 3).

‘Musculoskeletal headaches’ were listed in one cohort study [31], but Bath-related Headache [32], Pituitary Apoplexy [33] and Occipital Neuralgia [34] were not mentioned in cohort studies. A series on unruptured intracranial aneurysm proposed that expansion or thrombosis could lead to symptoms without rupture or subarachnoid haemorrhage [35].

#### Idiopathic thunderclap headache

Of the 459 people we identified with Idiopathic Thunderclap Headache there were a range of provoking activities, including sexual activity, bathing, and exertion (Table 4). In these 459 cases vasoconstriction was not sought or if sought, was not identified.

**Table 4:** 459 cases of idiopathic thunderclap headache, by study type and provocation

**Study type**	**Final diagnosis**	**N**		**Provocation**
Case	Bath-Related Thunderclap Headache [36-40]	16		Bathing
Series	Bath-Related Thunderclap Headache [32]	21		Bathing
Case	Weight lifters headache [41,42]	3		Exertion
Case	Primary Exertional [43-45]	7		Exertion
Cohort	Primary Exertional Headache [27]	17		Exertion
Case	Swimming headache followed by exertional and coital headache [46]	2		Exertion
Case	Primary Thunderclap Headache [47-49]*	13		Idiopathic
Cohort	Primary Thunderclap Headache [27-29,50]	200		Idiopathic
Case	Headache Associated with Sexual Activity [51-63]	42		Sexual activity
Cohort	Headache Associated with Sexual Activity ( [17,27,31]	48		Sexual activity
Series	Headache Associated with Sexual Activity [53,64,65]	86		Sexual activity
Case	Combined Benign Exertional and Benign Sexual Headache [52,66,67]	4		Sexual activity
**All**	**Total**	**459**		

*One case reported singing as a provocation of Thunderclap Headache [47].

#### Infection associated thunderclap headache

Cohort Studies identified infections in 7% of cases, and these were either unidentified infections or related to rhinosinusitis (Table 5). There has been at least one large series of aseptic meningitis where several cases presented with sudden and severe headache (Table 5). There are isolated case reports of sudden and severe headache which seem to be related to relatively rare infections such as Q-Fever and Erve Virus [68].

**Table 5:** 160 cases of infection associated sudden and severe headache, by study type

**Study type**	**Final diagnosis**	**N**
Cohort	Viral Illness [31]	41
Cohort	Rhinosinusitis [27,31]	36
Cohort	Aseptic meningitis [17,27-29,69]	27
Series	Aseptic meningitis [70]	24
Cohort	Headache attributed to systemic viral infection [27]	9
Case	Erve virus infection [71]	7
Cohort	Bacterial meningitis [27,31,69]	4
Case	Erve virus infection and migraine [71]	2
Case	*Acute Q-fever with cerebellar & meningeal involvement [72]	1
Case	*Bacterial meningitis secondary to a transethmoidal encephalocele [73]	1
Case	*Dengue haemorrhagic fever [74]	1
Case	Pneumococcal Meningitis, lower lobe pneumonia [75]	1
Cohort	*Acute Cranial Herpes Zoster [27]	1
Cohort	Herpes Encephalitis [28]	1
Case	Erve Virus Infection and Sinusitis [71]	1
Case	Persistent Primary Thunderclap Headache - related to Erve virus [76]	1
Case	*Subarachnoid Haemorrhage due to Borrelia burgdorferi-associated vascultis [77]	1
Case	Thunderclap headache secondary to complicated sinusitis [78]	1
	Subtotal cohort	119
	Subtotal case series	24
	Subtotal case reports	17

*Causes not mentioned in published Academic Reviews.

#### Cerebrovascular disease

We separated these cases into groups where there was no evidence of segmental arterial constriction non-vasoconstrictive aetiology (398 cases - Table 6) and vasoconstriction syndromes, (234 cases - Table 7).

**Table 6:** 398 cases of neurovascular thunderclap headaches, excluding vasoconstriction

**Study type**	**Final diagnosis**	**N**
	**Intravascular Air ( N = 2)**	
Case	*Air embolus [79]	1
Case	*Cerebral Venous Air Embolism following dental implanation [80]	1
	**Aneurysm ( N = 18)**	
Cohort	Unruptured Intracranial [27]	1
Cohort	Giant Aneurysm [28]	1
Series	Unruptured Intracranial Aneurysm [35]	7
Case	Unruptured Intracranial Aneurysm [48,81-83]	4
Case	*Recurrence and growth of previously coiled aneurysm [84]	2
Case	*Carotid artery aneurysm & carotid cavernous fistula [85]	1
Case	Unruptured Intracranial Aneurysm with Thrombosis [86]	1
Case	Carotid artery aneurysm thrombosis [87]	1
	**Cervicocephalic Arterial Dissection (N = 31)**	
Cohort	Carotid Artery Dissection [27]	1
Series	Vertebral Artery Dissection [88]	18
Case	Carotid Artery Dissection [89-91]	4
Case	*Basilar Artery Dissection (coital) [92]	2
Case	*Vertebral Artery Dissection with Reversible Cerebral Vasoconstriction Syndrome and RPLS [93]	1
Case	*Vertebral Artery Dissection, Subarachnoid Haemorrhage and PRES [93]	1
Case	Vertebral and Carotid Artery Dissection [94]	1
Case	*Vertebral Artery Dissection with Cerebellar Infarction [95]	1
Case	*Middle Cerebral Artery dissection [96]	1
Case	Vertebral Artery Dissection [97]	1
	**Intracranial Haemorrhage (N = 252)**	
Cohort	Subarachnoid Haemorrhage [17,27-29,31,69]	206
Cohort	Intracerebral Haemorrhage [17,28,29]	9
Cohort	Intracranial Haemorrhage [31]	1
Cohort	Subdural Haematoma [31]	1
Cohort	Haemorrhagic malignant glioma [28]	1
Series	Pre-truncal nonaneurysmal subarachnoid haemorrhage [98]	18
Case	Convexity Subarachnoid Haemorrhage [99]	3
Case	*Right convexity Subarachnoid Haemorrhage associated with right MCA atherosclerosis [99]	1
Case	*Simultaneous multiple brain haemorrhages associated with migraine, & cerebral amyloid angiopathy [100]	1
Case	Spontaneous Retroclival Haematoma [101]	1
Case	*Convexity Subarachnoid Haemorrhage with multiple brain abscesses [102]	1
Case	*Convexity Subarachnoid Haemorrhage with postpartum posterior encephalopathy [102]	1
Case	Convexity Subarachnoid Haemorrhage with vascultitis [102]	1
Case	*Trigeminal Haemorrhagic Inflammatory pseudotumour [103]	1
Case	Left thalamic haematoma [104]	1
Case	*Subarachnoid Haemorrhage secondary to ecchordosis physaliphora [105]	1
Case	*Headache secondary to diffuse subarachnoid density (blood and iohexol) on CT [106]	1
Case	Cerebellar Haemorrhage secondary to cerebellar angioma [107]	1
Case	Temporal Lobe Haematoma [107]	1
Case	Diffuse vasospasm after pretruncal nonaneurysmal subarachnoid haemorrhage [108]	1
	**Idiopathic (N = 4)**	
Cohort	Arteriovenous Malformation [28]	1
Case	Posterior reversible leucoencephalopathy [109]	1
Case	Thrombotic thrombocytopenia [110]	1
Case	Hypertensive Encephalopathy [111]	1
	**Non-Haemorrhagic Stroke or Cerebral Infarction or Ischaemia (N = 66)**	
Cohort	Stroke or Cerebral Infarction or Ischaemia [17,27-29,31]	56
Case	Ischaemic Cerebrovascular Disease [107]	3
Case	PICA Territory Infarction [112]	1
Case	Embolic Cerebellar Infarcts [113]	1
Case	Ischaemic stroke [114]	1
Case	Thunderclap Headache secondary to right semiovale infarction [115]	1
Case	Posterior cerebral artery infarction [116]	1
Case	Vertebral Artery Dissection with left lateral medullary infarction [117]	1
Case	*Occlusion of the posterior communicating artery [118]	1
	**Inflammatory Arteriopathy (N = 6)**	
Case	Cerebral Angiitis [119,120]	3
Case	*Vogt-Koyanagi-Harada Disease [121,122]	2
Case	Subarachnoid Haemorrhage secondary to Inflammatory Vasculitis [123]	1
	**Cerebral Venous Sinus Thrombosis (N = 19)**	
Cohort	Cerebral Venous Sinus Thrombosis [17,27,29]	3
Case	Cerebral Venous Sinus Thrombosis [124-129]	14
Case	Cerebral Venous Sinus Thrombosis with Intracerebral Haemorrhage [130]	1
Case	*Cerebral Venous Sinus Thrombosis with secondary Hydrocephalus [131]	1
		398

*Specific Aetiologies not mentioned in Academic reviews.

**Table 7:** 234 Cases of thunderclap headache with vasoconstriction

	**Vasoconstriction syndromes (N = 234)**	
Series	Reversible Cerebral Vasoconstriction Syndrome [132,133]	99
Series	Recurrent primary thunderclap headache and benign CNS angiopathy [134]	56
Case	Reversible Cerebral Vasoconstriction Syndrome [135-149]	18
Case	Reversible Cerebral Vasoconstriction Syndrome associated with Subarachnoid Haemorrhage [150]	6
Case	Postpartum Angiopathy with Reversible Posterior Leucoencephalopathy [151]	4
Case	Thunderclap headache with segmental intracerebral vasoconstriction [152]	3
Case	Cerebral Vasoconstriction Syndrome (Call-Fleming Syndrome) with ischaemic stroke [153]	3
Case	Reversible cerebral vasoconstriction syndrome with Subarachnoid Haemorrhage [123]	3
Case	Thunderclap headache with diffuse, multifocal, segmental, and reversible vasospasm [154]	2
Case	*Bilateral parietooccipital convexity Subarachnoid Haemorrhage with vasoconstriction [99]	1
Case	*Left cerebral convexity Subarachnoid Haemorrhage with vasoconstriction [99]	1
Case	Complicated Migraine associated with orgasmic cephalgia [155]	1
Case	severe migraine variant with segmental cerebral arterial narrowing and watershed infarction [156]	1
Case	Reversible Cerebral Vasoconstriction Syndrome with Cerebral Infarction [157]	1
Case	Recurrent thunderclap headache with reversible intracerebral vasospasm and stroke [158]	1
Case	Diffuse Cerebral Vasospasm - possibly ergotamine associated [159]	1
Case	Reversible Cerebral Vasoconstriction Syndrome with posterior leukoencephalopathy syndrome [160]	1
Case	Bathing headache with diffuse vasospasm with posterior leukoencephalopathy [36]	1
Case	Delayed Eclampsia with associated vasospasm [161]	1
Case	Cerebral Vasoconstriction with right MCA cerebral infarction [162]	1
Case	Thunderclap headache with posterior leukoencephalopathy syndrome [163]	1
Case	*Orgasmic headache with Transient Basilar Artery Vasospasm [164]	1
Case	Orgasmic headache and cerebral vasospasm [165]	1
Case	Thunderclap headache with cerebral vasospasm [166]	1
Case	Call-Fleming PostPartum Angiopathy in the Puerperium [167]	1
Case	Posterior leucoencephalopathy associated with vasospasm [168]	1
Case	Nonaneurysmal subarachnoid haemorrhage with secondary cerebral vasoconstriction [169]	1
Case	Call-Fleming syndrome [170]	1
Case	*Reversible Cerebral Vasoconstriction Syndrome following Spontaneous Intracranial Hypotension [171]	1
Case	Call-Fleming Syndrome [172]	1
Case	Left frontal intracerebral haemorrhage secondary to benign angiopathy of the central nervous system [173]	1
Case	Reversible Cerebral Vasoconstriction Syndrome [135]	1
Case	Cerebral Vasoconstriction Syndrome with Subarachnoid Haemorrhage [174]	2
Case	Convexity Subarachnoid Haemorrhage, with sulcal haematoma secondary to cerebral vasoconstriction [174]	1
Case	Reversible Cerebral Vasoconstriction Syndrome causing right putaminal haemorrhage [175]	1
Case	Unruptured Intracranial Aneurysm with vasoconstriction [176]	1
Case	Cocaine related Neurovascular Headache [177]	1
Case	*Late onset eclampsia with cortical blindness [178]	1
Case	Reversible Cerebral Vasoconstriction Syndrome associated with orgasmic headache [179]	1
Case	*Tacrolimus Encephalopathy [180]	1
Case	Headache associated with Postpartum Cerebral Angiopathy [181]	1
Case	Reversible posterior leucoencephalopathy associated with minimal change disease [182]	1
Case	Post partum cerbral angiopathy (Call fleming syndrome) [183]	1
Case	Eclamptic subarachnoid haemorrhage secondary to Post-partum cerebral angiopathy [184]	1
Case	*Recurrent PRES in a patient with sickle cell disease [185]	1
Case	Ipsilateral reversible cerebral vasoconstriction after Carotid Endarterectomy [186]	1
Case	Benign exertional and sexual headache with arterial spasm [187]	1
Case	Reversible cerebral vasoconstriction Syndrome associated oxymetazoline nasal spray [188]	1

*Specific Aetiologies not mentioned in Academic reviews.

A wide range of non-vasoconstrictive pathologies exist that could provoke sudden and severe headache, including all types of intracranial haemorrhage (subdural, subarachnoid, intracerebral and one single case of epidural haematoma as a retroclival haematoma), arterial dissection, venous sinus thrombosis, expanding or thrombosed berry aneurysm, venous thrombosis. Intravascular air was reported in isolated case reports only. It is possible that some of the cases of convexity or intraparenchymal haemorrhage were secondary to vasoconstrictive processes, but we were unable to confirm this from the published material.

Ischaemic stroke also features as an association of sudden and severe headache in cohort studies as well in individual case reports, but whether the infarction process is the cause of headache or whether the headache was a manifestation of an occult vasculopathy, such as a vasoconstriction syndrome, remains uncertain (Table 6).

We identified 234 cases where vasoconstriction was a plausible or proven mechanism for headache. It was not always reported whether vasoconstriction was reversible, as this would require reporting of normal follow-up imaging. Vasoconstriction syndromes could be associated with a range of factors, specifically post-partum states [151], cocaine use [177], spontaneous intracranial hypotension [171], sickle-cell disease [185] and posterior leucoencephalopathy [160] (Table 7).

#### Non-vascular, neurological causes

5% of published cases had a non-vascular, neurological aetiology, including intracranial hypotension [189], pneumocephalus [190], pituitary apoplexy [33], other cerebral neoplasms [191], including colloid cyst of the third ventricle [192]. Psychological presentations [193] and post-seizure headache [27] were amongst reported causes of sudden and severe headache (Table 8).

**Table 8:** 119 Neurological, non-vascular cases of thunderclap headache

**Study type**	**Final diagnosis**	**N of cases**
	**Intracranial Air ( N = 6)**	
Case	*Pneumocephalus associated with frontal pneumosinus dilatans [194]	1
Case	*Pneumocephalus [195-199]	5
**40**	**CSF Pressure Disorders (N = 40)**	
Cohort	Headache due to low CSF pressure [27]	14
Cohort	Idiopathic Intracranial Hypertension [27]	1
Cohort	*Cerebral Oedema [17,29]	2
Case	Spontaneous Intracranial Hypotension [79,189,200-206]	16
Case	Third Ventricle Colloid Cyst [192,207]	2
Case	*Adult aqueductal stenosis [208]	1
Case	Intracranial hypotension secondary to retained spinal needle [79]	1
Case	Intraventricular arachnoid cyst [209]	1
Case	Cerebellar tonsillar herniation [210]	1
Case	Hydrocephalus secondary to third ventricle dermoid cyst [211]	1
**3**	**Neuro-Inflammatory Disorders (N = 3)**	
Cohort	*HaNDL Syndrome [27]	1
Cohort	*Ocular Inflammatory Disorder [27]	1
Case	*Apoplectic headache secondary to Multiple Sclerosis [212]	1
**4**	**Chemical Meningitis (N = 4)**	
Case	*Rathke's cleft cyst [213,214]	2
Case	*Ruptured spinal dermoid tumour [215]	1
Case	*Ruptured arachnoid cyst into subdural space [216]	1
**7**	**Intracranial Neoplasm (N = 7)**	
Cohort	Neoplasm [27,31,69]	6
Case	*Nonhaemorrhagic Anaplastic Oligodendroglioma [191]	1
**43**	**Pituitary Apoplexy (N = 43)**	
Case	Pituitary Apoplexy [33,217-223]	8
Case	*Sphenoid sinus epidermoid cyst presenting as pituitary apoplexy [224]	1
Series	Pituitary Apoplexy [225]	34
**1**	**Psychiatric Disorder (N = 1)**	
Case	*Transitional Interpersonality Thunderclap Headache [193]	1
**15**	**Other (N = 15)**	
Cohort	*Post Seizure Headache [27]	2
Series	Occipital Neuralgia [34]	12
Case	*Hyperventilation Induced headaches [226]	1
		119

*Specific Aetiologies not mentioned in Academic reviews.

#### Systemic illnesses

A small number of cases (13 in total) were reported in association with non-neurological, non-cerebrovascular causes, the most prominent of which was acute myocardial infarction (Table 9).

**Table 9:** 13 Cases with systemic illness in association with sudden and severe headache

**Study type**	**Final diagnosis**	**N**
**Case**		**7**
Case	Acute Myocardial Infarction [26,227-232]	8
Case	Aortic Dissection [233-235]	3
Case	Thunderclap Headache Secondary to Phaeochromocytoma [236,237]	2
Case	Acute Puerpural headache & hypertension [238]	1
Case	Acute Intracranial Hypertension due to Occlusion of the Brachiocephalic vein [239]	1
Case	Coital Headaches induced by Amiodarone [240]	1
Case	Coital headache secondary to thyrotoxicosis (l [51])	1
**Cohort**		**6**
Cohort	Musculoskeletal [234]	5
Cohort	Alcohol Induced Headache [234]	4
Cohort	Analgesic Overuse Headache [234]	3
Cohort	Arterial Hypertension [234]	1
Cohort	Amphetamine Misuse [234]	1
Cohort	Triptan overdose	1

### Comparison of causes identified in academic reviews versus systematic review

Of the causes not reported in academic reviews, and identified in our review, intracranial air, either intravascular or spontaneous pneumocephalus was the most frequently omitted cause (Table 8).

Case reports did identify potentially serious isolated causes of sudden and severe headache, specifically Aortic Dissection and Myocardial Infarction. Ecchordosis Physaliphora is a notochord related remnant which can cause intracranial haemorrhage, which was fatal in a published case report [105]. Psychologically mediated causes of Sudden and Severe Headache were also identified (Transitional Interpersonality Disorder and Hyperventilation Attacks). Perhaps surprisingly, there was no publication of Munchausen’s Syndrome as a cause of sudden and severe headache.

### Non-English articles

The full text of a total of 29 non-English publications were examined once we had completed the main review of English Language publications - 27 case reports from 22 publications [241-263], and 599 cases from 8 case series [264-271]. The single large case series from Japan described 562 people with thunderclap headache, normal CT Brain and normal visually inspected CSF. Of these 562 people 52 berry aneurysms were identified at angiography, 46 of which went to surgery, 8 of which had evidence at surgery of recent haemorrhage, 4 of which had abnormal CSF cell count or protein levels [264,265].

There were 4 non-English language cohort studies [271-274], which did not identify any additional causes of abrupt, severe headache. In the 6 non-English academic review articles [20-25], ice-cream headache, exploding head syndrome and aortic coarctation were listed as potential causes of acute headache, although these causes were not identified in our own literature search, and exploding head syndrome was excluded as it is an intracranial noise rather than a headache.

Unique cases from non-English case report literature included Headache Associated with Sexual Activity associated with a Frontal Meningioma [246] or Intraventricular Arachnoid Cyst [244], Subarachnoid Haemorrhage secondary to Vertebral Artery Dissection [257], Convexity Subarachnoid Haemorrhage following Spinal Anaesthesia [249], Acute Hydrocephalus secondary to Chiari Malformation [259], and Pneumocephalus following inadvertent dural puncture at Epidural Anaesthestic [263].

4 cases of aneurysmal subarachnoid haemorrhage associated with normal CT Brain and normal appearing CSF (by visual inspection, not by spectrophotometry) were identified from a large case series from Japan [264,265]. The 4 non-English Language Cohort Studies did not identify causes that were not already identified from English language articles.

From the non-English academic reviews, Hypnic Headache, Cluster Headache, Exploding Head Syndrome, Ice-cream Headache and Aortic Coarctation were listed as causes of sudden and severe headache. It was not possible to get more clinical information on the published case of aortic coarctation which was included as part of a larger cohort.

## Discussion

We present a comprehensive review of the literature on Sudden AND Severe Headache up to September 2009.

From our search, we estimate that we have identified 98% of all published causes of Sudden AND Severe Headache in the English Literature.

The literature on sudden and severe headache has some significant weaknesses, yet allows us to make some interesting observations on this potentially serious symptom and how information is reported in medical literature.

Prospective, longitudinal studies of unselected cases are crucial for determining accurate frequencies of incidence or cause. The only estimate of population-based incidence is from Sweden, at 43 cases per 100,000 adults per annum, which was estimated from a sub-set of the population in their study [17]. This figure is important as it is the only one that can inform service planning for the assessment of sudden and severe headache across a large population.

Our understanding of causes of sudden and severe headache is evolving, yet we do not have a precise knowledge of trends or causes of this symptom in a population over time. An emerging theme are the cerebral vasoconstriction syndromes which are attracting a lot more attention in the medical literature and since our literature review have been the subject of several major reviews [275,276], yet this cause was not described in any of the large cohort studies we found in our systematic review.

In cohort studies, where neurologists assigned diagnosis, headaches of unspecified cause, uncertain cause or a primary headache accounted for the majority of cases in unselected populations. This is contrary to hospital based clinical practice where a diagnosis of migraine, tension-type headache or cluster headache would hardly ever be used in the context of an abrupt onset of severe headache. Sudden and severe headache which remains unexplained following appropriate investigation would normally be called Primary or Idiopathic Thunderclap Headache. Reasons for this large number of unexplained or uncertain headaches remain obscure.

Another seemingly important cause of headache in a population is cervicogenic headache, which was identified in about 4% of adults in the Vaga Study [277]. The cohort studies identified a small number of ‘musculoskeletal’ related headaches [31] and there was one case series of occipital neuralgia presenting acutely to hospital [34], but otherwise this common headache is unreported as causing sudden and severe headache, yet seems a plausible candidate for many of the ‘uncertain’ or ‘unclassified’ cases.

Intracranial Hypotension is an important cause which featured in both cohort and case-based studies - so is both common enough to see in practice, and its treatable nature makes it reportable.

An up to date population-based study could make an important contribution to our current knowledge of sudden and severe headache, specifically the true incidence and prognosis of vasoconstriction syndromes, and better assessment of the clinical features and prognosis of those with ‘uncertain’ or ‘unclassifiable’ headaches.

Cases identified from series are important, as they aggregate information on relatively low frequency conditions as a reference for other clinicians. However, they distort the overall frequency of causes of headaches in our review eg pituitary apoplexy was not reported in the major cohorts. Nonetheless, accumulating cases of specific sub-types can greatly inform clinical practice and generate research hypotheses. An example of this is Headache Associated with Sexual Activity where a recent case series from Taiwan has identified the Reversible Cerebral Vasoconstriction Syndrome in almost 2/3rds of cases [278].

By their nature, case reports will focus on the esoteric or unusual, but our review has highlighted some important associations including systemic conditions like Aortic Dissection [233,234,279,280], Cardiac Cephalalgia [26,227-229,231,232,281-283], Phaeochromocytoma [236,237], serious infections like Dengue Haemorrhagic Fever [74] and Q-Fever [72], as well as Erve Virus [68,71]. Rare vasculopathies such as Vogt-Kayanagi-Harada Disease [121,122], or cerebral vasculitis [119] do appear on the differential diagnosis. Another important presentation, identified in case reports only, was basilar artery dissection manifesting as a coital headache [92].

Cerebral vasculitis is commonly mentioned in discussions about sudden and severe headache, but in numeric terms, we only identified 6 cases of an ‘inflammatory arteriopathy’ compared to 234 cases of a likely vasoconstriction.

### Vasoconstriction syndromes

There is evidence that many cases of otherwise unexplained Sudden AND Severe Headache, particularly coital headache, are actually due to Reversible Cerebral Vasoconstriction [278,284], and that pain is mediated by sympathetic innervated cerebral arteries. A recent systematic review of 214 cases of Reversible Cerebral Vasoconstriction Syndrome found 94% cases presented with thunderclap headache, which was often recurrent [276]. Our review identified 234 cases where vasoconstriction was the likely mechanism of headache (Table 9). Of these 234 cases, 37 (16%) had complications such as posterior leucoencephalopathy (11 cases, 5%), ischaemic stroke (7 cases, 3%) or haemorrhage (19 cases, 8%).

It is interesting to observe that low CSF pressure states have been accompanied by reversible cerebral vasoconstriction on one [171] if not 2 occasions [135]. This raises the intriguing possibility that Lumbar Puncture may not always be advisable when investigating a Thunderclap Headache if cerebral vasoconstriction is present. However, there is insufficient data to guide clinical practice at present.

Our review still supports the concept that Vasoconstriction Syndromes are probably part of a spectrum of disorders with an isolated sudden and severe headache at one end and multiple headaches and serious neurovascular complications at the other [278,284].

### Publication bias

One of our review’s objectives was to identify all causes as a prelude to writing a management guideline for Thunderclap Headache, and we were aware of relevant academic review articles [3-9]. Our systematic review did identify additional causes of sudden AND severe headache, such as Aortic Dissection and Pneumocephalus that were not specifically mentioned in these review articles. This is not to say that academic reviewers had no knowledge of these cases, but may merely reflect editorial practice. In fact 2 academic reviews stated search criteria [3,18]. However, some causes of Thunderclap Headache are more commonly listed than others eg spontaneous retroclival haematoma, even when there has only ever been one solitary case published [101].

Our review collates a large number of published causes of sudden and severe headache and may serve as a benchmark for clinicians and researchers alike, as we estimate that our review may be 98% complete. Evidence based practice requires systematic critique of evidence, and our review is novel for applying this method to a list of causation. Medical lists are ubiquitous in medical education and clinical practice guidelines. The value of ‘lists’ is that information can be memorised and disseminated easily, but by being non-systematic they may perpetuate error as well as fact.

### Limitations of this review and subsequent publications

Since the review completed, there have been some additional cases added to the medical literature, including spontaneous spinal epidural haematoma [285], and several more associations of the Reversible Cerebral Vasoconstriction Syndrome such as carotid glomus tumour [286] and Takayasu’s Arteritis [287]. There has also been a major academic review on Thunderclap Headache published in 2013 [288] which did not identify additional cases to those mentioned in our own review [275]. We continue to monitor publications on thunderclap headache, but this paper will be limited to cases published by September 2009.

A potential criticism of this work is that we did not use the International Headache Society Classification of Headache Disorders. However, retrospective use of the criteria was not possible from the majority of published articles where criteria were not specified. If we had attempted to apply IHS criteria we may have created further error by assigning a classification without full information. Nonetheless, the objective of our review was to try and determine the full range of potential diagnoses that might emerge in someone who has presented for the assessment of a sudden and severe headache. We feel we have been as comprehensive and accurate as the published literature allows.

### Using this data in clinical practice

In clinical practice, the differential diagnosis is central to organising diagnostic tests. The diagnostic evaluation is a screening process for target diseases. We can now argue that in a patient presenting with a sudden and severe headache that we have now created a definitive list of possible causes for sudden and severe headache i.e. we have quantified the possible diagnoses that have made it to the published literature, and that this list is arguably 98% complete.

The cohort studies provide the relative frequency with which target diseases might occur in practice, and it is common sense to look for these in the first instance. The case series and case reports identify cases that illustrate unusual mechanisms for sudden and severe headache - for example myocardial infarction or pituitary apoplexy.

Clinicians looking to manage people with sudden and severe headache should now consider this list as a definitive reference, and if they think they identified an additional cause they can rapidly verify whether their case is unique, and therefore meriting publication.

### Future research

As our paper has shown that there is a difference between the causes of sudden and severe headache published in academic reviews and those identified from a systematic review, it would be important to maintain this list on an ongoing basis as a reference point for clinicians and researchers.

Amongst potential problems in current medical publishing practice is that articles run out of date, and systematic reviews need updating. At present, the Cochrane Collaboration do not provide for systematic reviews of causation, yet this information is important in clinical practice when trying to reach a diagnosis.

There is also a need to do an up to date prospective, population based study of sudden and severe headache with a specific emphasis on identifying the frequency of vasoconstriction syndromes, the long term prognosis of cases in whom aneurysmal subarachnoid haemorrhage is excluded, and examining cases where a clinical diagnosis remains elusive, even after examination by a neurologist.

## Conclusions

This paper presents what we regard as a definitive list of associations of Sudden and Severe Headache. This list is important as it demonstrates that the differential diagnosis of sudden and severe headache is substantial. Our review confirms that a wide range of diseases and provoking circumstances are associated with sudden and severe headache, specifically, haemorrhage, thrombosis, dissection, arterial expansion, extremes of intracranial pressure, infection, inflammation and even intracranial air. In large unselected cohorts the majority of cases are assigned a primary headache diagnosis or have a cause of headache that does not meet any specific diagnostic criteria.

New cases of sudden and severe headache associated with conditions which are not represented in our list should be considered for publication in the medical literature as novel causes.

## Competing interests

RF has received unrestricted educational grants to fund attendance at European Neurological Society meeting in Athens 2004 (value <1000GBP), The International Headache Society Meeting in Stockholm 2007 (value <1000GBP) and The American Headache Society Meeting Boston June 2008 (value <2000GBP), The EHMTIC meeting London 2012 (value <1000GBP) from Janssen-Cilag and Allergan pharmaceutical companies. The Neurology Department in which RF practices received an unrestricted educational grant from Cyberonics for an economic analysis of vagus nerve stimulators in 2007 (value 7000GBP). RF has received an honorarium for contributions at an educational meeting sponsored by Allergan in Dublin November 2013 (1065GBP). RF runs a website called severe-headache-expert.com which earns income from advertising. HN no conflicts of interest. ED no conflicts of interest.

## Authors’ contributions

RF initiated and designed the review, screened the articles, assisted with data abstraction, data analysis, co-wrote and edited and approved the final manuscript, and is ultimately accountable for this work. HN designed the systematic review and obtained articles from external sources. ED abstracted data from identified articles, summarized and analysed data, wrote initial version of manuscript and approved final version for submission. All authors read and approved the final manuscript.
